# Laparoscopic Heller myotomy with Dor fundoplication for achalasia: an outcome in a tertiary health center of Nepal

**DOI:** 10.3389/fsurg.2026.1678605

**Published:** 2026-01-22

**Authors:** Kunal Bikram Deo, Parbatraj Regmi, Narendra Pandit, Barurendra Raj Yogi, Bed Prakash Sah, Ulav Budhathoki, Shailesh Adhikary

**Affiliations:** 1 Department of Gastrointestinal Surgery, B P Koirala Institute of Health Sciences, Dharan, Nepal; 2 Department of Surgery, BP Koirala Institute of Health Sciences, Dharan, Nepal; 3 Department of Surgical Gastroenterology, Birat Medical College and Teaching Hospital, Biratnagar, Nepal

**Keywords:** achalasia cardia, Heller myotomy, laparoscopic surgery, laparoscopy, outcome

## Abstract

**Introduction:**

Laparoscopic Heller Myotomy with Dor fundoplication is the most effective therapeutic option for Achalasia cardia, with fewer complications. We present the outcomes of this procedure with long-term follow-up in patients with Achalasia cardia.

**Methods:**

A single institution prospectively maintained data of Laparoscopic Heller Myotomy with Dor fundoplication between January 2014 and January 2024 was reviewed. Eckardt scores at three-time points (preoperative, 3-month, and long-term follow-up) were used to assess treatment efficacy.

**Results:**

A total of 16 patients had a median age of 34 years. Megaesophagus was observed in 8 (50%) patients, and 5 patients had sigmoid esophagus. The mean operative time was 162 ± 41 min. The mean myotomy length was 6.1 cm and 2.19 cm, respectively, for the esophagus and the stomach. Following the surgery, there was significant improvement in the Eckardt score from a median preoperative score of 9 (5–12) to a median postoperative score of 2(0–4) in 3 months (*p* = 0.001) and a median Eckardt score of 1.5 (0–3) in long-term follow-up (*P* < 0.001). The median long-term follow-up was 32 months (12–60 months). Overall, two treatment failure was observed, and one required endoscopic balloon dilatation. The gastroesophageal reflux (uncomplicated) was observed in 5 (31.2%) patients. The symptoms were mild, and none had reflux-related complications at the last follow-up.

**Conclusions:**

LHM provides immediate and durable symptomatic relief with fewer complications.

## Introduction

Achalasia Cardia is a primary motility disorder of the esophagus characterized by failed lower esophageal sphincter (LES) relaxation during swallowing with an absence of coordinated esophageal contractions (1). These features are well documented by HRM of the esophagus, the current gold standard for the diagnosis (2). As HRM is not widely available in developing nations, traditional tools like barium swallow and esophagogastroscopy are still useful for diagnosis. Typical symptoms of dysphagia, regurgitation, chest pain, and weight loss are utilized in the Eckardt symptom (ES) score, a well-validated method widely used to assess achalasia cardia symptom severity and systematically evaluate treatment efficacy as well (3).

All therapeutic approaches, like graded pneumatic dilation, laparoscopic Heller myotomy (LHM), and peroral endoscopic myotomy (POEM), aim at reducing LES pressure. LHM with partial fundoplication and POEM are established surgical approaches with the highest success rate. While POEM offers an attractive approach with no surgical scar, it has a high learning curve as well as high rates of gastroesophageal reflux (4). LHM with partial fundoplication is the gold standard for achalasia cardia with low reflux rates (1). Studies with long-term follow-up of >10 years have shown sustained relief in 80%-90% of patients (5, 6).

**Figure 1:**
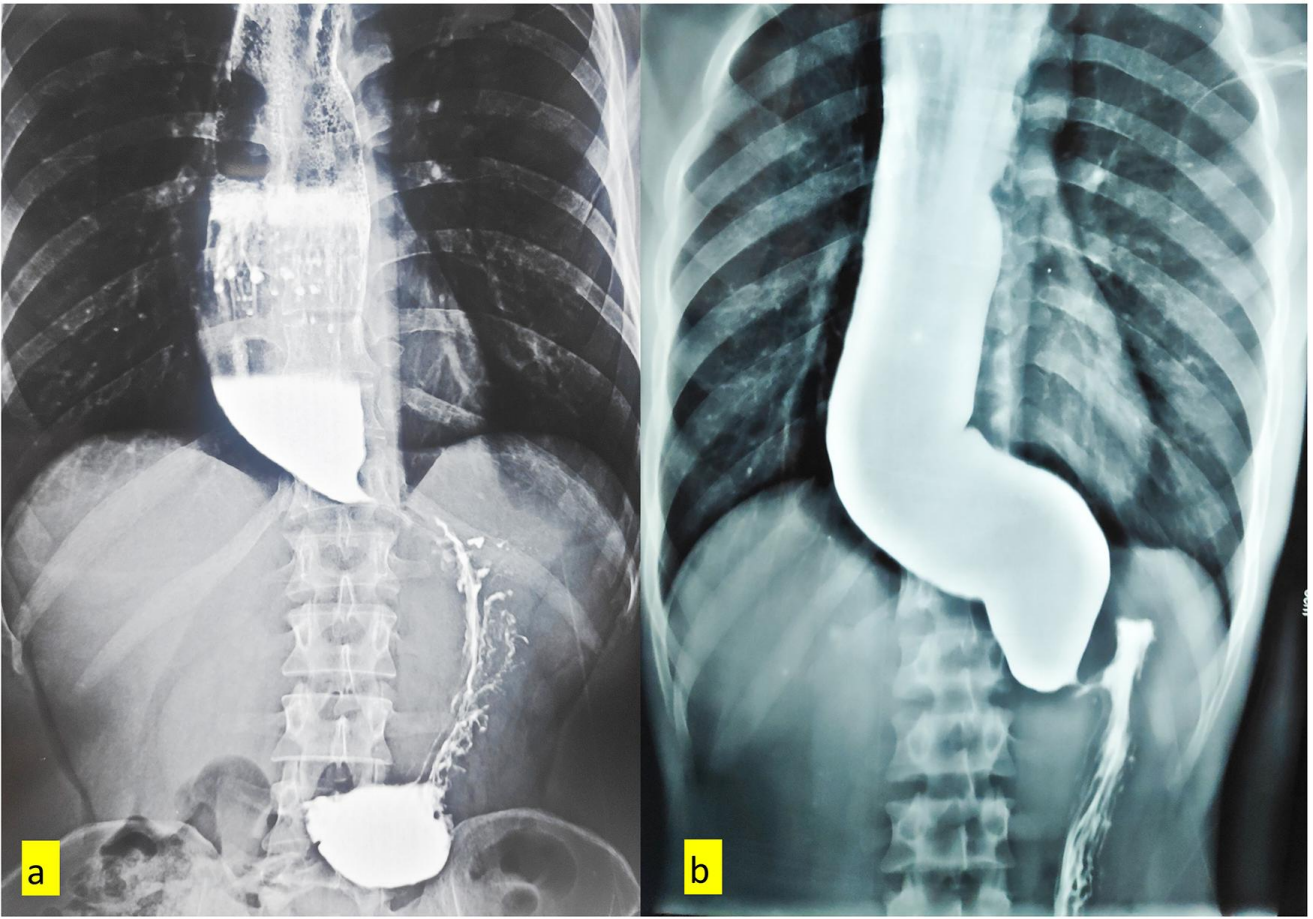
**(a)** contrast esophagogram showing megaesophagus. **(b)** Contrast esophagogram showing Sigmoid esophagus.

The delay in diagnosis, unawareness amongst the patients, poor health care access, and underdiagnosis in developing nations often lead to the advanced presentation of achalasia cardia, like megaesophagus and sigmoid esophagus. (Figure 1) Although the treatment success rates of myotomy in these advanced diseases are low, LHM remains the most effective initial management (7). Our institute has also adopted LHM with Dor fundoplication as the standard treatment for achalasia cardia. With the usual logistic challenges of developing nations and patients presenting with advanced disease, we aim to present the feasibility, postoperative outcomes, and long-term efficacy of LHM with Dor fundoplication for achalasia cardia using the ES score.

## Materials and methods

This study was ethically approved by the institutional review committee (2843/024) and conforms to the provisions of the Declaration of Helsinki. A prospectively maintained database of all patients diagnosed with achalasia cardia in the Department of Gastrointestinal Surgery from January 2014 to January 2024 was reviewed. The diagnosis of achalasia cardia was achieved with a combination of symptoms, esophagogastroscopy, and contrast esophagogram. Esophageal manometry was used selectively in doubtful cases, especially in patients with early achalasia cardia due to logistical reasons. Contrast-enhanced computed tomography of the chest and abdomen was used selectively to rule out secondary achalasia. Megaesophagus in achalasia cardia was defined as esophageal dilatation >6 cm, and if it had a sigmoid shape, it was labelled as sigmoid esophagus (2). All patients undergoing LHM with Dor Fundoplication for achalasia cardia were included. The department's expert gastrointestinal surgeons performed all surgeries. The patient's clinico-demographic parameters, diagnostic modalities, operative details, and postoperative outcomes were reviewed. The objective assessment of the severity of achalasia cardia was done using the Eckardt scoring (ES) system (3). (Table 1) The ES score was calculated at three time points: 1) Preoperative. 2) around a 3-month follow-up. 3) last follow-up. The ES score was calculated with proper explanation to patients in their native language, mediated by the surgeons at the bedside and during outpatient follow-up. The outcome of the surgery was assessed by comparing postoperative ES at outpatient follow-up with the preoperative ES score. The treatment failure was defined as an ES score >3, clinical symptom progression, and the need for any reintervention (8). The morbidity and mortality were measured with the Clavien-Dindo score (9). Long-term outcomes, such as reflux, were assessed based on the patient's subjective complaints.

**Table 1:** ECKARDT symptom score.

Score	Symptom			
	Weight loss	Dysphagia	Retrosternal pain	Regurgitation
0	None	None	None	None
1	<5 kg	Occasionally	Occasionally	Occasionally
2	5–10 kg	Daily	Daily	Daily
3	>10 kg	Each Meal	Each Meal	Each Meal

### Surgical technique

**Figure 2:**
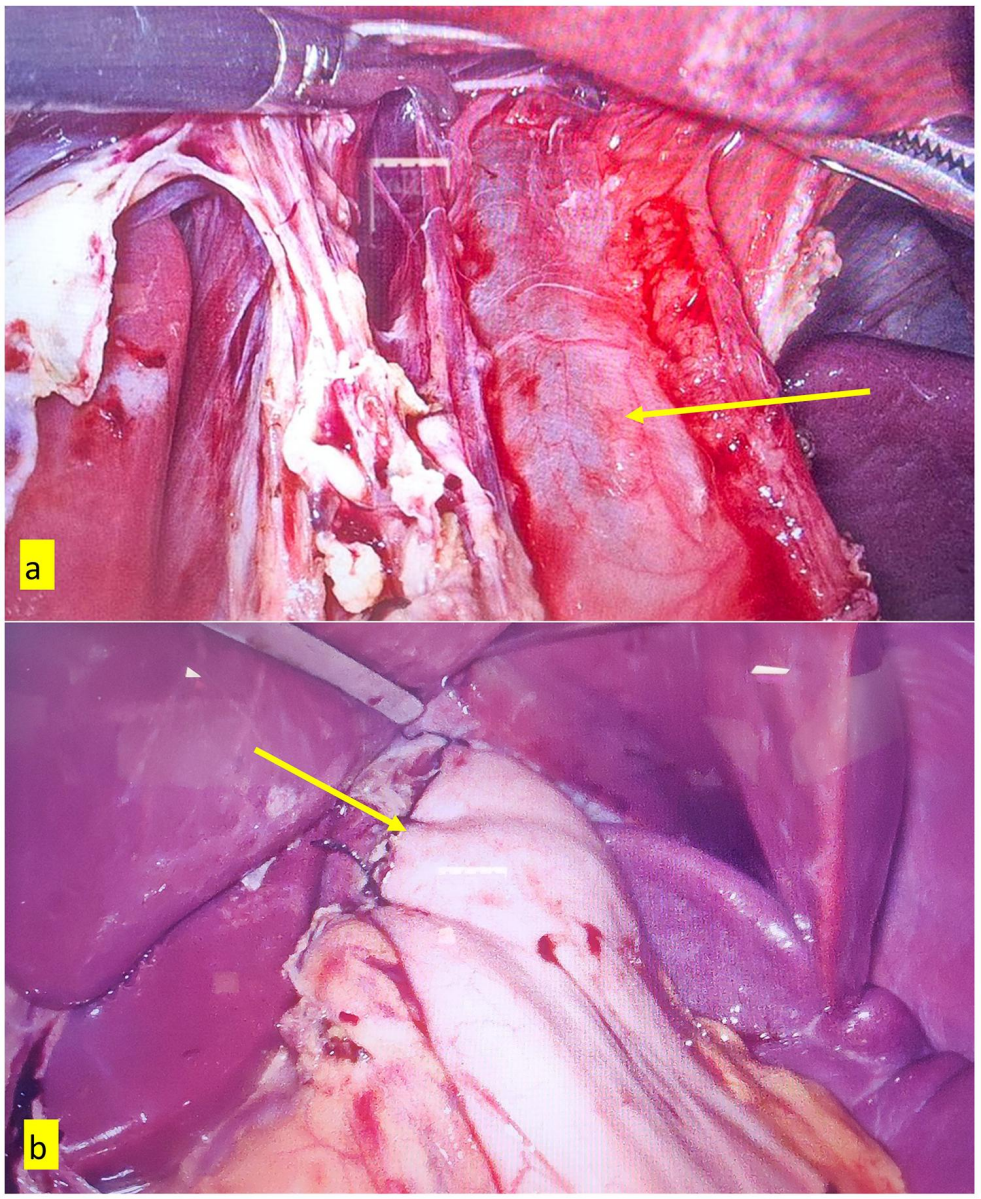
**(a)** arrow in the intraoperative image showing completed esophageal myotomy **(b)** arrow in the intraoperative image showing completed Dor fundoplication.

Laparoscopic Heller myotomy was performed using the standard technique. We additionally mobilized the lower mediastinal esophagus to achieve adequate myotomy. We did not use any energy source to perform myotomy. Myotomy was perform Esophageal myotomy was performed up to 5–7 cm in the right anterolateral side and extended to 2–3 cm on the stomach. The completeness of myotomy and mucosal injury was evaluated by thorough inspection and an air insufflation test after the instillation of methylene blue dye via a proximally placed NG tube. The Dor fundoplication was performed in all patients. (Figure 2) Nasogastric was removed, and oral feeding started routinely in first postoperative day in an uneventful surgery. Postoperative contrast swallow study was done selectively.

### Statistical analysis

All data were analyzed with SPSS version 22 for statistical analysis. The descriptive analysis was performed by calculating percentages, means, and medians wherever appropriate. The baseline ES score was compared with the ES scores at 3 months and long-term follow-up using the Wilcoxon signed-rank tests. The median ES scores between the groups were compared using the Mann–Whitney U test. All tests of association were two-sided, and *p-*value*s* below 0.05 were considered statistically significant.

## Results

A total of 21 cases of achalasia cardia were planned for surgery, out of which 4 patients refused surgery for various reasons, and one underwent open Heller myotomy in the COVID-19 period, where the safety of the laparoscopic procedure was uncertain. Finally, the clinical, perioperative, and follow-up details of 16 patients were analyzed in the study. (Table 2) The median age of the patients during surgery was 34 years (range: 20–84 years), and 5/16 (31.3%) patients were female. The median duration of dysphagia was 30 months (range: 3–60 months). Two (12.5%) patients had a history of recurrent pneumonia. One patient presented with anemia, sepsis, and retention esophagitis. Megaesophagus (esophageal diameter >6 cm) was observed in 8 (50%) patients, and five (31.3%) patients had sigmoid esophagus. (Figure 1) Esophageal HRM was performed on 4 patients, with two patients each in the Chicago type I and II categories. One patient had prior endoscopic balloon dilatation. All patients underwent LHM with Dor fundoplication. (Figure 2) The mean operative time was 162 ± 41 min. The mean myotomy length was 6.1 ± 0.9 cm for the esophagus and 2.19 ± 0.4 cm for the stomach. (Figure 2) One patient had an esophageal mucosal injury detected and repaired intraoperatively with uneventful recovery. The median blood loss was 50 mL (range: 30–200 mL). The median postoperative hospital stay was 3 days (range: 2–15 days). One patient had morbidity due to aspiration pneumonia and sepsis, managed with antibiotics and critical care management. There was no operative mortality.

**Table 2:** Descriptive statistics of patients undergoing laparoscopic Heller myotomy for achalasia cardia.

Patient characteristics	Data
Patients	16
Median Age (range) (years)	34.5 (20–84)
Sex	
Male	11 (68.8%)
Female	5 (31.3%)
Dor Fundoplication	16 (100%)
Median duration of dysphagia (months)	30 (3–60)
History of pneumonia	2 (12.5%)
Megaesophagus	8 (50.0%)
Sigmoid esophagus	5 (31.3%)
Mean Operative time	162 ± 40.7
Median Blood loss (range) (mL)	50 (30–200)
Myotomy	
Esophagus	6.1 ± (0.9)
Stomach	2.19 ± 0.4
Median length hospital stay (days)	3.0 (2–15)
Median follow-up (range)(months)	32 (12–60)
Patients with	
1–2 years follow-up	3 (18.8%)
2–5 years follow-up	10 (62.5%)
5 years or more follow-up	3 (18.8%)
Postoperative intervention	
Balloon Dilatation	1 (6.2%)

### Outcomes

The median preoperative ES score was 9 (range: 5–12). (Table 3) Dysphagia was the most common symptom, with a median sub-score of 3. The median sub-score for regurgitation was 2.5, for retrosternal chest pain was 1, and for weight loss was 2. At a follow-up of around 3 months, there was a significant improvement in ES symptom scores from a baseline median of 9–2 (*p* = 0.001). The median ES score in the early postoperative period, at 3 months, was 2 (range: 0–4). The symptom score further improved in long-term follow-up with a median ES score of 1.5 (range: 0–3) compared to a baseline of 9 (range: 5–7) (*p* < 0.001). One patient had an ES score of 4, and another patient had an ES score of 3 with a dysphagia sub-score of 3. The median duration of follow-up was 32 months (range: 12–60 months). The one-year follow-up was achieved in 3 (18.8%) patients, 2–4 years in 10 (62.5%) patients, and 5 years in 3 (18.8%) patients.

**Table 3:** Eckardt symptom score at three-time intervals.

Eckardt score	Preoperative baseline	3-month post-LHM	W value*	*p-*value*	Long-term follow-up	*W* value**	*p-*value**
Overall median ES score	9 (5–12)	2 (0–4)	−3.41	0.001	1.5 (0–3)	−3.534	<0.001
Dysphagia, Median	3 (1–3)	0 (0–3)	−3.391	0.001	0 (0–3)	−3.493	<0.001
Weight loss, median	2 (1–3)	0 (0–1)	−3.376	0.001	0 (0–1)	−3.573	<0.001
Regurgitation, median	2.5 (1–3)	0 (0–1)	−3.568	0.001	0 (0–1)	−3.568	<0.001
Retrosternal pain, median	1 (0–3)	0 (0–2)	−2.326	0.20	0 (0–2)	−2.178	0.29

The Wilcoxon signed-rank test was used to compare the Eckardt score between various time frames.

* *p* and *W* value of the comparison between “preoperative baseline” with and 3-month post-LHM.

** *p* and *W* value of the comparison between “preoperative baseline” with long-term follow-up.

The patient with an ES score of 4 in a 3-month follow-up had a dysphagia sub-score of 3 and a regurgitation score of 1. The endoscopic evaluation showed a lax esophagogastric junction. Subsequently, over 3 years patient's dysphagia resolved completely, but had persistent symptoms of retrosternal pain, and regurgitation with weight loss of <5 kg, giving the Eckardt score of 3. The dysphagia in a patient with an ES score of 3 months improved in subsequent follow-up. Another patient had a progression of dysphagia from a sub-score of 1 at 3 months to 3 in 30 months of follow-up and subsequently managed with balloon dilatation. Thus, two treatment failure was observed, out of which one improved spontaneously, and another patient required reintervention. The remaining patients experienced an excellent recovery. Gastroesophageal reflux was present in 5 (31.2%) patients in long-term follow-up. The proton pump inhibitor was required in 4 (25%) patients. The symptoms were mild, and none had reflux-related complications at the last follow-up.

The Subgroup analysis of the megaesophagus patients was done and compared with non-megaesophagus patients. The baseline median ES score of 10 (range: 8–12) in patients with megaesophagus was significantly higher compared to the median ES score of 8 (range: IQR: 5–9) in non-megaesophagus patients. However, the median Eckardt Score significantly decreased from baseline of 10 (range: 8–12) (median IQR) to median of 2 (range: 1–3) in early postoperative follow-up at 3 months (*p* = 0.011) and further to median of 2 (range: 1–2) on long-term follow-up (*p* = 0.011) in patients with megaesophagus. This trend was observed in both megaesophagus and non-megaesophagus, with no significant difference in the decrease in median ES score post surgery between these groups (Table 4).

**Table 4:** Comparison of the eckardt scores between patients with megaesophagus and patients with no megaesophagus.

Esophagus type	Median Eckardt score (range)						
	Preoperative baseline	3-month post-LHM	W value*	*p-*value*	Long-term follow-up	*W* value**	*p-*value**
Megaesophagus	10 (8–12)	2 (1–3)	−2.38	0.017	2 (1–2)	−2.55	0.011
Non-megaesophagus	8 (5–9)	1.5 (0–4)	−2.53	0.011	1 (0–3)	−2.53	0.011
*p-*value***	0.007	0.46			0.57		

The Wilcoxon signed-rank test was used to compare the Eckardt score between various time frames.

* *p* and *W* value of the comparison between “preoperative baseline” with and 3-month post-LHM.

** *p* and *W* value of the comparison between “preoperative baseline” with long-term follow-up.

*** *p* value after Mann–Whitney U test comparing the Eckardt score between the megaesophagus and Non-Megaesophagus group.

## Discussion

This study represents the first evaluation of perioperative and long-term outcomes of LHM with Dor fundoplication in achalasia cardia from Nepal, with a case series (10) of three patients published earlier by another institution. Our study demonstrates that LHM with Dor fundoplication is feasible and effective. Although we observed two treatment failures as per the operational definition, only one patient required reintervention, and another improved in subsequent follow-up with conservative management.

Laparoscopic Heller myotomy has emerged as the gold standard surgical management of achalasia cardia. The large-scale studies by Rosemurgy et al. (11). and Zaninotto et al. (6) encompassing over 500 and 400 patients, respectively, have reported treatment efficacy exceeding 90%. The safety, feasibility, and excellent outcomes of LHM are well documented in multiple studies (12–15), contributing to its widespread adoption. The standard myotomy length ranges from 4 to 6 cm in the esophagus and 2–3 cm in the stomach. However, to minimize the risk of incomplete myotomy, particularly in cases of potential long-segment achalasia cardia, we maintained an extended esophageal myotomy of 6–7 cm, given the selective use of HRM. The role of extended myotomy was further underscored by Oelschlager et al. (16), who demonstrated that a 3 cm gastric myotomy resulted in significantly lower LES pressure and reduced dysphagia compared to a shorter (1–1.5 cm) myotomy.

HRM plays a pivotal diagnostic role in achalasia cardia, facilitating classification into three subtypes per the Chicago classification (17), thereby guiding myotomy length and prognostic outcomes (2). However, its utility in the megaesophagus and sigmoid esophagus remains uncertain. Due to logistical constraints and financial limitations in our patient population, HRM was selectively employed. Moreover, 50% of our cohort presented with megaesophagus or sigmoid esophagus, where HRM findings are less reliable.

Peroral endoscopic myotomy (POEM) represents a minimally invasive alternative, unconstrained by diaphragmatic anatomy. Its ability to tailor myotomy length makes it particularly advantageous for type III achalasia cardia. A multicenter randomized controlled trial (RCT) by Werner et al. (4). established POEM's non-inferiority to LHM with Dor fundoplication, with overall clinical efficacy exceeding 90% (18). Nevertheless, POEM's steep learning curve and limited accessibility in resource-constrained settings restrict its widespread adoption. Additionally, POEM is associated with higher reflux rates (44% at 2 years) compared to LHM with Dor fundoplication (29%) (4).

The disruption of circular muscle fibers at the gastroesophageal junction (GEJ) during myotomy raises concerns about postoperative gastroesophageal reflux disease (GERD). The prospective RCT by Richards et al. (19). demonstrated that pathological reflux rates decreased from 47% (LHM alone) to 9% with adjunctive Dor fundoplication. Long-term follow-up (median 11.8 years) further confirmed no significant difference in dysphagia scores, alleviating concerns about dysphagia following partial fundoplication (20). While the choice between Dor and Toupet fundoplication remains surgeon-dependent, multiple studies (21–23) report comparable efficacy, safety, and long-term GERD outcomes. We preferred the Dor technique due to its simpler anterior approach, mucosal coverage benefits, and concerns that Toupet fundoplication may angulate the GEJ in the sigmoid esophagus, impairing esophageal emptying. The 360^0^ Nissen fundoplication was largely abandoned due to a 15% incidence of dysphagia without additional reflux benefits (24). However, a recent study by Yetişir et al. (25). showed that Extended myotomy with Nissen fundoplication provides superior outcomes in achalasia cardia treatment, including lower recurrence rates, fewer GERD symptoms, and higher patient satisfaction compared to Dor fundoplication groups. The elevated reflux rates observed in our study may be attributed to a small sample size and subjective GERD assessment, as objective reflux scoring was logistically infeasible.

The management of end-stage achalasia cardia (megaesophagus or sigmoid esophagus) remains contentious, with some advocating esophagectomy (26) and others endorsing myotomy as initial therapy (27). Half of our samples exhibited end-stage disease, defined by the 2018 ISDE guidelines as severe dilation or sigmoid morphology resulting from untreated or recurrent achalasia cardia (2). End-stage progression occurs in 5%–25% of cases (28) with massive dilatation predisposing to myotomy failure due to inefficient esophageal emptying and elevated malignancy risk. A study by Tassi et al. (29) (*n* = 583, median follow-up 147 months) reported a squamous cell carcinoma incidence of 1.61/1,000 person-years, with a cumulative 13% risk at 56.34 years. The sigmoid esophagus conferred a 17.6-fold increased cancer risk (95% CI: 4.13–75.43). Despite these risks, myotomy achieves >89% efficacy in the sigmoid esophagus (30) and a recent meta-analysis (7) reported a 76% probability of good/excellent outcomes (95% CI: 0.703–0.812; *p* < 0.01). Our study similarly demonstrated favorable outcomes in end-stage disease, supporting myotomy as a first-line therapy to avoid esophagectomy-related morbidity. Esophagectomy should be reserved for refractory cases or malignancies, with long-term surveillance mandated due to cancer risk.

This study highlights the outcomes of LHM in a resource-limited setting, demonstrating its safety and efficacy despite challenges. One notable morbidity occurred in a patient with a sigmoid esophagus, who developed aspiration pneumonia postoperatively. This case was complicated by preexisting retention esophagitis, anemia, and occult pneumonia, likely exacerbated by aspiration. Such complications underscore the importance of meticulous perioperative care, particularly in patients with advanced disease.

Iatrogenic mucosal injury remains a concerning complication of LHM, with reported rates ranging from 2.1% to 23.9% in the literature (31, 32). Given the unavailability of intraoperative endoscopy, the standard tool for ensuring myotomy adequacy and detecting mucosal injuries, we employed alternative measures, including diligent visual inspection, methylene blue dye testing, and air insufflation. Additionally, to minimize thermal injury, we avoided energy sources during myotomy. Initial bleeding during blunt myotomy was successfully controlled with gauze compression, and only one case of mucosal injury was identified and repaired intraoperatively.

### Limitations

This study has a few limitations inherent to its retrospective design, including small sample size, single-center study, selection bias, potential recall bias, and selective use via HRM due to logistical reasons and short follow-up duration. GERD in our study was solely assessed by subjective symptoms due to the unavailability of pH studies, which may have led to under- or over-evaluation of reflux symptoms. The lack of intraoperative endoscopy, a gold standard for confirming myotomy completeness, was a significant constraint. Additionally, most patients presented at advanced disease stages, likely due to financial barriers and limited access to tertiary healthcare.

Despite these challenges, our outcomes align with global standards, reinforcing the feasibility of LHM even in resource-limited settings. The study population, drawn from the eastern region of Nepal, reflects the realities of delayed presentation yet demonstrates that satisfactory outcomes are achievable with experienced surgical teams.

## Conclusion

In conclusion, LHM remains a safe, effective, and durable treatment for achalasia cardia, even in settings with constrained resources. Our findings support its role as the gold standard intervention for achalasia cardia when performed by experienced foregut surgeons. Future prospective studies with larger cohorts, longer follow-ups, and standardized objective assessments (e.g., routine postoperative endoscopy and pH monitoring) would further validate these results.

## Data Availability

The raw data supporting the conclusions of this article will be made available by the authors, without undue reservation.
